# Microfluidics in cardiovascular disease research: state of the art and future outlook

**DOI:** 10.1038/s41378-021-00245-2

**Published:** 2021-03-03

**Authors:** Qingming Ma, Haixia Ma, Fenglan Xu, Xinyu Wang, Wentao Sun

**Affiliations:** 1 School of Pharmacy, Qingdao University, Qingdao, 266071 China; 2 Center for Prenatal Diagnosis, Zibo Maternal and Child Health Care Hospital, Zibo, 255000 China; 3 Department of Clinical Pharmacy, The Affiliated Hospital of Jiangsu University, Jiangsu University, Zhenjiang, 212001 China; 4 Institute of Thermal Science and Technology, Shandong University, Jinan, 250061 China; 5 Center for Basic Medical Research, TEDA International Cardiovascular Hospital, Chinese Academy of Medical Sciences & School of Medicine, Nankai University, Tianjin, 300457 China

**Keywords:** Materials science, Engineering

## Abstract

Due to extremely severe morbidity and mortality worldwide, it is worth achieving a more in-depth and comprehensive understanding of cardiovascular diseases. Tremendous effort has been made to replicate the cardiovascular system and investigate the pathogenesis, diagnosis and treatment of cardiovascular diseases. Microfluidics can be used as a versatile primary strategy to achieve a holistic picture of cardiovascular disease. Here, a brief review of the application of microfluidics in comprehensive cardiovascular disease research is presented, with specific discussions of the characteristics of microfluidics for investigating cardiovascular diseases integrally, including the study of pathogenetic mechanisms, the development of accurate diagnostic methods and the establishment of therapeutic treatments. Investigations of critical pathogenetic mechanisms for typical cardiovascular diseases by microfluidic-based organ-on-a-chip are categorized and reviewed, followed by a detailed summary of microfluidic-based accurate diagnostic methods. Microfluidic-assisted cardiovascular drug evaluation and screening as well as the fabrication of novel delivery vehicles are also reviewed. Finally, the challenges with and outlook on further advancing the use of microfluidics technology in cardiovascular disease research are highlighted and discussed.

## Introduction

According to the World Health Organization (WHO), cardiovascular diseases (CVDs) have become the most prevalent noncommunicable diseases globally and are responsible for an estimated 17.9 million deaths each year^1,2^. CVDs are a group of cardiovascular system disorders, including hypertension, stroke, hypercholesterolemia, diabetes, coronary heart disease, chronic kidney disease, peripheral arterial disease and vascular dementia^3^. Specifically, the cardiovascular system is composed of three basic components, the heart, blood and vasculature^4^, with the heart generating the driving pressure to pump blood through the vasculature to circulate throughout the body^5^. Each component contains multiple functional elements^6^. For instance, the heart integrates cardiomyocytes, a fibrous skeleton and cellular electrical conductance pathways^7^, while blood comprises erythrocytes, immune cells, platelets and acellular plasma where multiple important protein pathways occur, including fibrin polymerization, complement activation and fibrinolysis^8^. Moreover, the vasculature contains several key constituents, such as the endothelium, that control the transport of active biomolecules and regulate inflammation, blood pressure and immune cell trafficking^9,10^. Dysfunction of these elements, along with other risk factors such as hereditary susceptibility, high misdiagnosis rates and a lack of clearly defined risk assessment criteria, lead to cardiovascular system disorders and result in CVDs with various clinical symptoms^11,12^. Due to its severe morbidity and mortality globally, comprehensive investigations of CVDs have attracted scientific interest, and many achievements have been obtained^13–15^.

Comprehensive research on CVDs can be divided into three major aspects: the study of pathogenetic mechanisms, the development of accurate diagnostic methods and the establishment of therapeutic treatments^16,17^. Among these aspects, pathogenesis studies play a central role since they can not only provide physiological and pathological information for fundamental CVD research but also be further used as the research basis for diagnosis and treatments and therefore deserve prioritized investigation. Diagnosis is also critical since CVDs occur abruptly and progress rapidly; therefore, timely diagnosis and assessment of progression is extremely important for clinical treatment. Moreover, therapeutic treatment is the core to battle CVDs that can alleviate symptoms and decrease the overall risk of death^18,19^. Hence, achieving a holistic picture of CVD research requires comprehensive investigation of these three aspects, and strategies with the capability to integrally investigate CVDs covering all three aspects are highly desired.

Microfluidics, a science and technology that involves processing small (10^–9^ to 10^–18^ liters) volumes of fluids in microchannels with dimensions of tens to hundreds of micrometers, has recently been recognized as having inherent characteristics particularly well suited to modeling the cardiovascular system and can therefore be used as a versatile primary strategy in achieving comprehensive CVD research ranging from pathogenesis studies to diagnosis and treatments^20,21^.

It is relatively straightforward to utilize microfluidics as the primary strategy to facilitate the holistic investigation of CVDs^22–27^. It is worth noting that both cell-based and noncell-based microfluidics have been applied in CVD research. In particular, noncell-based microfluidics are mainly used to develop microchannels modified with photodiodes and specific antigens for the detection of various biomarkers to facilitate the diagnosis of CVDs^28–39^. Only a few noncell-based microfluidic platforms have been used to develop a hemodynamic microenvironment with precise control over blood flow to investigate pathogenesis, such as the formation mechanism of thrombosis^40^. In contrast, cell-based microfluidics are applied more widely in all three major aspects of CVD research^41–43^. Cells are either embedded in microfluidic channels and used directly or patterned and cultured for further use^44,45^. With cell-based microfluidics, artificial 3D tissue-like cardiovascular architectures can be achieved, and “human-on-a-chip” architectures can be obtained, whereby the vasculature and heart cultures can mimic complex interactions between organs and body systems and provide suitable environments for investigating the pathogenesis, diagnosis and treatments of CVDs in a more efficient manner^46–48^.

**Fig. 1:**
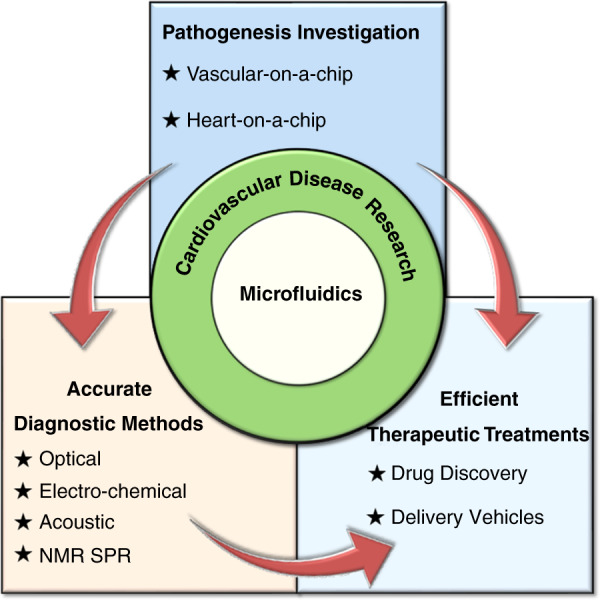
An overview of applying microfluidics in cardiovascular disease research. The key advantages of microfluidics in CVDs research are illustrated, including pathogenesis investigation and the development of accurate diagnostic methods and efficient therapeutic treatments.

Here, we summarize recent progress in the use of microfluidics in comprehensive CVD research, ranging from investigating the underlying pathogenesis to developing accurate diagnoses for efficient therapeutic treatments (Fig. 1). Representative examples are reviewed with expanded discussions analyzing the findings, highlighting the beauty of fundamental research and envisioning therapeutic applications. At the end, challenges and perspectives on further advancing microfluidics in comprehensive CVD research are proposed and discussed.

## Microfluidics for investigating the pathogenesis of CVDs

The occurrence and development of cardiovascular disease is a complex process involving multiple pathogenic factors and regulated by various mechanisms^49^. Uncovering the critical underlying pathogenesis of disease is the basis for developing diagnostic and therapeutic methods. Microfluidic technologies can offer visible microchannels with versatile geometric designs that can simulate the complex structural architectures of microvascular networks^50^. They can offer precise control over the flow conditions of miniscule amounts of fluids in microchannels, which benefits the imitation of the hemodynamics of cardiovascular blood flow^51^. In addition, the surface properties of microchannels can be elaborately modified to perform delicate studies that replicate intricate cardiovascular biophysical interactions^52,53^. Therefore, microfluidics can be used to create organs-on-a-chip that simulate the activities, mechanics and physiological responses of the cardiovascular system and can be used as platforms for studying the pathogenesis of CVDs^54,55^. Specifically, microfluidic-based cardiovascular-mimetic organs-on-a-chip can construct a hemodynamic microenvironment that is close to the in vivo state of the cardiovascular system, provide precise individual control over each pathogenic factor, and regulate cellular behaviors as well as cell-cell interactions under blood flow conditions, thus allowing for novel opportunities to directly study cardiovascular physiology, pathology, and pharmacology^56,57^. Hence, microfluidic-based cardiovascular-mimetic organs-on-a-chip, such as vasculature-on-a-chip and heart-on-a-chip, have been widely applied as powerful in vitro platforms for revealing the critical pathogenesis of various cardiovascular diseases (Table 1).

**Table 1:** Examples of microfluidic-based cardiovascular-mimetic organs-on-a-chip for investigation of the pathogenesis of different CVDs.

Disease model	Microfluidic building materials	Chip type	Cell incorporated	External stimulation involved	Ref.
Arteriovenous thrombosis	PDMS bonded on glass	Vascular-on-a-chip	Platelet	Mechanical	40
Arteriovenous thrombosis	PDMS on silicon wafers	Vascular-on-a-chip	Human endothelium cells & platelet	Mechanical	46
Arteriovenous thrombosis	PDMS	Vascular-on-a-chip	Human endothelium cells & platelet	Mechanical	48
Atherosclerosis	PDMS bonded on glass	Vascular-on-a-chip	Endothelial cells	Mechanical	44
Atherosclerosis	PDMS	Vascular-on-a-chip	Endothelial cells	Electrical	67
Myocardial infarction	PDMS bonded on glass	Heart-on-a-chip	Myocardium cells	Mechanical	70
Myocardial infarction	PDMS bonded on glass	Heart-on-a-chip	Myocardium cells & skeletal muscle cells	Mechanical	41
Heart failure	PDMS	Heart-on-a-chip	Cardiac cells	Electrical	42
Disordered intercellular electromechanical transduction	PDMS bonded on glass	Heart-on-a-chip	Myocardium cells	Electrical	68

## Microfluidic-based vascular-on-a-chip

Hierarchical vascular structures and functional damage are the most important pathological bases of CVDs. Microfluidics has been applied to facilitate the establishment of in vitro vasculature-on-a-chip that can be used as suitable platforms for investigating the pathogenesis of various vascular diseases^58,59^.

**Fig. 2:**
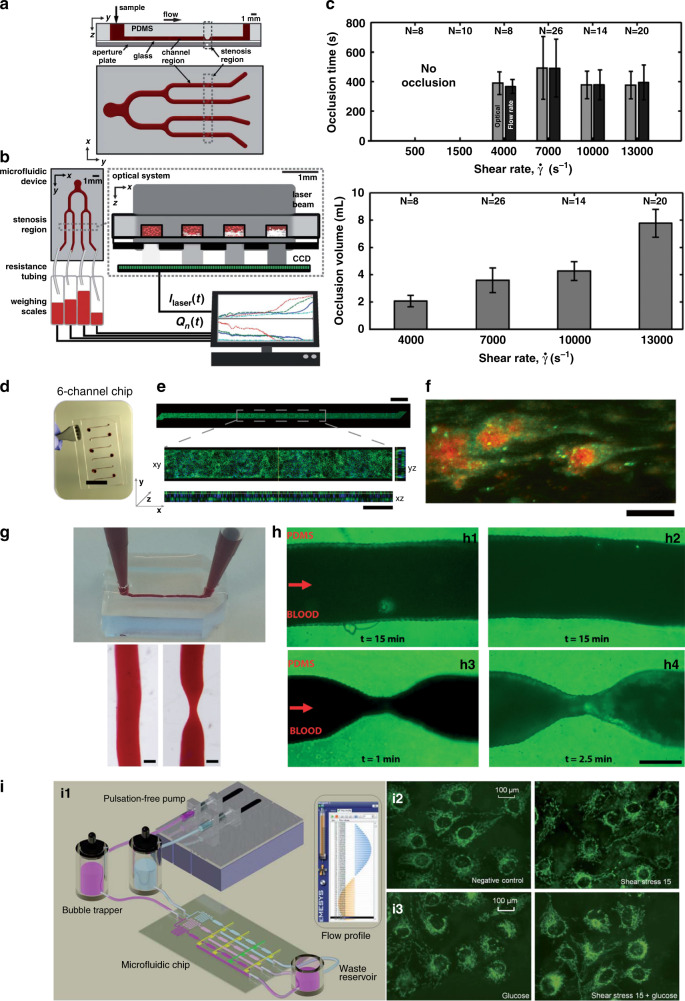
Microfluidic-based vascular chips to study the pathogenesis of thrombosis. **a**–**c** Microfluidic-based vascular system to investigate the relationships of platelet aggregation time and blood volume with shear rates. **d**, **e** HUVEC-modified microfluidic chip, scale bars: 15 mm (**d**) and 200 μm (**e**). **f** Formation of fibrin (green) along with platelet aggregates (red), scale bar: 20 μm. **g** Microfluidic chips perfused with blood, scale bars: 370 μm. **h** Formation of thrombosis in stenotic geometry, scale bars: 200 μm. **i** Vasculature-on-a-chip to mimic the blood flow in vessels (i1); Mitochondrial morphology of endothelial cells without (i2) or with (i3) glucose under different shear stresses. **a**–**c** Reproduced with permission^40^. Copyright 2012, The Royal Society of Chemistry. **d**–**f** Reproduced with permission^46^. Copyright 2016, Springer Nature. **g**, Reproduced with permission^48^. Copyright 2017, The Royal Society of Chemistry. **i** Reproduced with permission^44^. Copyright 2019, The Royal Society of Chemistry.

Arteriovenous thrombosis is the pathological formation of platelet aggregates that occlude blood flow and is one of the major causes of cardiovascular events. Many microfluidic-based vascular models have been developed to screen and study predisposing factors.^46,60–63^ For instance, a polydimethylsiloxane (PDMS) microfluidic chip integrated with an optical system has been established for simultaneously measuring platelet aggregation at different initial shear rates within four stenotic channels in label-free whole blood, as illustrated schematically in Fig. 2a, b^40^. Requisite shear rates spanning physiological to pathological flow conditions (500–13,000 s^−1^) can be achieved in the chip. The aggregation of platelets can be measured in real time by using an optical system, as shown in Fig. 2b. The results show that the amount of blood volume producing occlusion depends on the blood shear rate, as illustrated by the plots in Fig. 2c. The results indicate that a high shear rate of blood flow would accelerate platelet aggregation and therefore increase the incidence of thrombosis. However, this platform only investigates the pathogenesis from the physical perspective, and bioactive components such as vascular endothelial cells should also be taken into account when designing microfluidic-based vascular chips to study the pathogenesis of thrombosis^64^. As illustrated in Fig. 2d, e, a microfluidic chip with channels chemically lined by human endothelial cells (HUVECs) was developed. HUVECs fixed on the surface of the channels can still secrete adhesion molecules and induce platelet aggregation when triggered by inflammatory factors. These chips can be used to study the role of vascular endothelium in the formation mechanism of thrombosis in more biomimetic environments that are superior to that of the described platform that only investigates this pathogenesis from the physical point of view^46^. Moreover, the formation mechanism of thrombosis also relies strongly on 3D vessel geometry and local blood flow patterns. Microfluidic chips with well-designed structures that closely mimic vascular architectures found in both healthy and stenotic blood vessels have been established, as illustrated in Fig. 2g^48^. The microfluidic channels are also modified with HUVECs and then perfused with human whole blood with fluorescently labeled platelets at a physiological shear rate. There is no sign of thrombosis in the microfluidic chips with healthy geometries after 15 minutes of perfusion (Fig. 2 h1–h2), while obvious thrombosis can be seen in the chips with stenotic geometries, as shown in Fig. 2 (h3–h4), indicating that the narrow vessel geometry and tight local blood flow patterns indeed give rise to platelet aggregation and trigger blood occlusion. Considering the abovementioned examples together, a full view of applying microfluidics in investigating the pathogenesis of arteriovenous thrombosis can be obtained that can be further used as a basis to facilitate the development of diagnostic and treatment methods for thrombosis.

Atherosclerosis is one of the most common forms of CVD and stems from chronic inflammation of the endothelium. Various works utilizing microfluidic-based vascular chips as models to study the pathogenesis of atherosclerosis have been reported^65^. For example, a hemodynamic microfluidic-based vasculature-on-a-chip was designed to investigate the effect of pulsatile shear stresses and glucose concentrations on the intracellular reactive oxygen species (ROS) level and the mitochondrial morphology of endothelial cells, as illustrated in Fig. 2i. ROS levels are elevated when cells are subjected to blood pulsatility and high glucose concentrations, as demonstrated by the fluorescence microscopic images and plots in Fig. 2i. The increased cellular ROS level would further induce morphological changes in mitochondria from filamentous reticular networks to diffuse and short fragments, triggering the pathology of atherosclerosis^44^. Moreover, a refined microfluidic-based early-stage atherosclerosis model that can simultaneously model hemodynamic factors such as flow shear stress and periodic stretching as well as pathological factors such as high glucose concentration, high cholesterol level and various proinflammatory factors has also been developed to further investigate the mechanism of atherosclerosis^66^. The abovementioned risk factors increase cellular ROS production and reduce vascular endothelial cadherin expression, thereby promoting the occurrence of atherosclerosis. In addition to the risk factor-induced ROS level elevation, the disordered endothelial microenvironment is also critical to the induction of atherosclerosis. For instance, a microfluidic-based vascular chip was designed to monitor endothelial permeability using integrated electrodes and end-point characterization of the endothelium through immunostaining. The results demonstrate that endothelial monolayer permeability and adhesion protein expression change in response to oscillatory shear stress frequency caused by disturbed flow. These changes are found to be significant above certain frequencies (such as 1 Hz normalized to 10 dyne/cm^2^), suggesting that a frequency threshold must be reached to elicit an endothelial response and induce the initiation of atherosclerosis^67^. In conclusion, by applying microfluidics, the pathogenesis of atherosclerosis can be well interpreted in vitro, which offers great possibilities for conducting diagnosis and treatments of atherosclerosis in clinical trials.

## Microfluidic-based heart-on-a-chip

In addition to vascular-on-a-chip, microfluidics has also been applied to establish in vitro heart models by manipulating heart tissue to create morphological, electrophysiological and contractile microenvironments that are closer to physiological states. The developed heart-on-a-chip can be used as an in vitro platform to investigate the pathogenesis of cardiac diseases^68,69^.

**Fig. 3:**
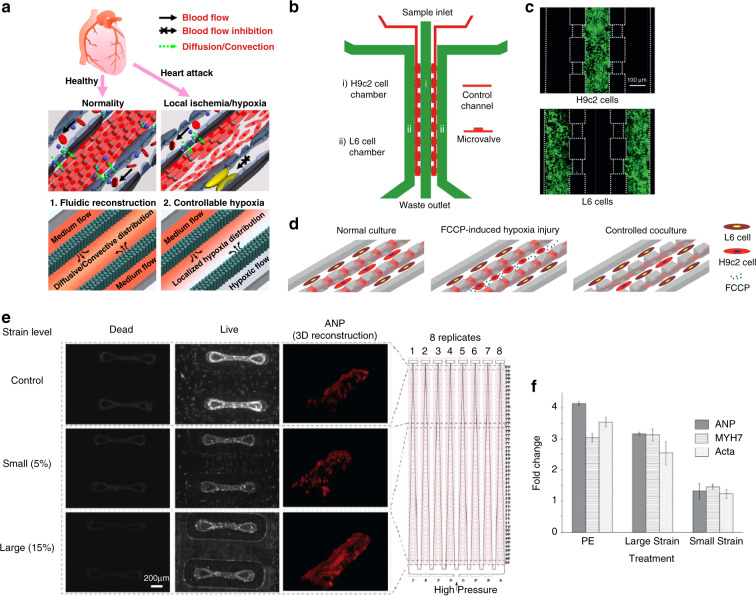
Microfluidic-based heart chips to study the pathogenesis of CVDs. **a** Microfluidic device for studying myocardial hypoxia. **b** Microfluidic-based chip monitoring of skeletal myoblast transplantation. **c** CLSM images of H9c2 and L6 cells cultured with high viability. **d** Schematics illustrating the on-chip study procedures. **e** CLSM images of cell viability and ANP expression. **f** Plots illustrating the upregulated expression levels of ANP, MHC-β and ACTA1 under cardiac hypertrophy. **a** Reproduced with permission^70^. Copyright 2017, American Chemical Society. **b**, **c**, **d** Reproduced with permission^41^. Copyright 2018, The Royal Society of Chemistry. **e**, **f** Reproduced with permission^42^. Copyright 2019, The Royal Society of Chemistry.

Myocardial infarction is one of the most common acute and severe types of CVD, and its occurrence is typically caused by continuous hypoxia of the myocardium. To dynamically study hypoxia-induced myocardial injury in a microenvironment-controllable manner, a microfluidic-based heart model was developed and used to investigate the response of myocardial cells under hypoxic conditions, as illustrated in Fig. 3a^70^. Specifically, the developed heart-on-a-chip offer precise control over blood flow in the microchannels, and myocardium H9c2 cells are seeded and cultured to form artificial myocardial tissue with well-organized structures. By introducing a chemical hypoxia reagent, an in situ spatial hypoxia condition can be created in the chips. The results show that hypoxia can directly lead to observable cell shrinkage, disintegration of the cytoskeleton, loss of mitochondrial membrane potential and obvious activation of caspase-3, which demonstrates that hypoxia can induce significant pro-apoptotic effects and myocardial infarction. Moreover, a microfluidic-based heart model was designed to coculture skeletal muscle cells and myocardial cells under hypoxic conditions, as illustrated in Fig. 3b^41^. The interactions between skeletal L6 myoblasts and hypoxia-injured H9c2 myocardium cells were investigated (Fig. 3c). The results show that skeletal myoblasts repair hypoxia-injured myocardial cells through direct cell-to-cell interactions. This platform can offer new perspectives to treat myocardial infarction without any medication, and the principle can inspire new treatment methods for other CVDs.

Heart failure is the end-stage manifestation and the major cause of death for CVDs and is mainly caused by volume overload-induced cardiac hypertrophy. To more directly investigate its pathological mechanism, a pneumatic microfluidic-based heart model that enables robust manipulation of cardiac cells has been developed^42^. Heterotypic and homotypic cardiac tissues generated in the device are pneumatically loaded in a range of regimes, with real-time on-chip analysis of tissue phenotypes. In the developed cardiac hypertrophy model, the expression levels of atrial natriuretic peptide (ANP), cardiac myosin heavy chain beta (MHC-β) and alpha skeletal muscle actin (ACTA1) increase significantly, as shown by the confocal laser scanning microscopic (CLSM) images and plots in Fig. 3e, f, indicating that the disordered gene expression of myocardial cells under cardiac hypertrophy is closely related to the progression of heart failure^71,72^. In addition, another microfluidic-based heart model was developed that allows H9c2 cardiomyoblast cells cultured under desired hydraulic pressure to represent hemodynamic stress to cardiomyocytes in cardiac hypertrophy^73^. When the developed model is subjected to 170 mm Hg hydraulic pressure, the expression level of ANP is significantly increased, further demonstrating the relationship between the disordered gene expression of cardiomyoblast cells and the progression of cardiac hypertrophy. Moreover, the increased ANP expression can be quantified and used as an effective indicator of heart failure etiologies. In general, by using microfluidic-based heart models, the pathogenesis of heart failure can be investigated more directly, and new therapeutic entry points can be offered^74–76^.

Overall, the application of microfluidic-based reductionist models in studying the pathogenesis of CVDs has achieved significant progress, which can offer solid foundations for the development of diagnostic and treatment methods. However, there is still much room left for microfluidic-mediated pathogenesis studies, and the use of more intricate models may be a helpful complement. Specifically, most of the models currently in use are either reductionist vascular or heart-on-a-chip models; therefore, developing intricate models such as integrated cardiovascular organoids is challenging and would offer great possibilities to study the pathogenesis of CVDs in a more comprehensive manner^77^. For instance, by using microfluidic-based 3D bioprinting technology, endothelial cells can be directly bioprinted within microfibrous hydrogel scaffolds to form a layer of confluent endothelium. Then, 3D endothelial networks can be seeded with cardiomyocytes to facilitate the formation of integrated cardiovascular organoids^78^. The developed integrated cardiovascular organoids can offer spontaneous and synchronous contraction and can be used as a suitable platform to mimic hierarchical cardiovascular structures and functional damage to facilitate pathogenesis studies. Furthermore, this intricate cardiovascular model can also be used in personalized drug screening to mitigate drug-induced cardiovascular toxicity and improve treatment efficacy.

## Microfluidics for developing accurate diagnostic methods for CVDs

CVDs are diseases that occur abruptly and progress rapidly; thus, timely diagnosis and assessment of the progression of CVDs is extremely important for clinical treatment, especially for point-of-care (POC). With the help of the increasing understanding of the pathogenesis of CVDs, biomarkers are recognized as the most commonly used diagnostic indicators to assess the physiological, pathogenic and pharmacological processes of CVDs. Representative biomarkers applicable for efficient diagnosis, such as C-reactive protein (CRP), B-type natriuretic peptide (BNP), low-density lipoprotein (LDL), and hydrogen peroxide, should have characteristics such as high specificity, easy accessibility, high stability and long plasma half-life^79^. However, the current clinical technologies for diagnosing CVDs based on biomarkers have problems such as high detection cost, large sample consumption and tedious operation steps, which largely hamper their application in the POC of CVDs. Due to the inherent characteristics of microfluidics, such as the decreased volume of liquid required and the ease of modification of the surface properties of microchannels by bioactives and cells, microfluidics is well suited for initiating delicate studies that replicate the intricate cardiovascular biophysical interactions and can therefore achieve diagnosis with low sample consumption, high sensitivity and automated analysis processes^52,53^. In addition, microfluidics can also be integrated with various thermal, irradiant and electrical detectors, therefore facilitating automation of the detection and subsequent analysis processes (Table 2).

**Table 2:** Summary of representative microfluidic-based diagnostic methods for CVD biomarkers.

Technique integrated	Microfluidic building materials	Detected biomarkers	Limitation of detection	Assay time	Sample volume
Field-Effect Transistor-based sensor	PDMS on epoxy substrate	CRP, BNP, cTnI, fibrinogen	CRP (0.1 mg/L), BNP (50 pg/mL), cTnI (1 pg/mL), fibrinogen (0.1 mg/mL)	5 min	4 μL^28^
Immunofluorescence	PDMS bonded on glass	CRP	1.4 nM	6 min	40 μL^29^
Immunofluorescence combined with cleavable tag immunoassay (CTI) and micellar electrokinetic chromatography (MEKC)	PDMS	CK-MB, cTnT, cTnI, Myo	CK-MB (3 ng/mL), cTnT (25 pg/mL), cTnI (2 ng/mL), Myo (5 ng/mL)	50 s	500 μL^30^
Chemiluminescence immunoassay	PDMS bonded on glass	cTnI	0.027 ng/mL	30 s	100 μL^31^
Fluorescence Resonance Energy Transfer	PDMS	cTnI	50 nM	10 min	20 μL^32^
Surface Enhanced Raman Scattering	PDMS in wax module	miR-29a	47 pg/μL	5 min	40 μL^33^
Electrochemical immunoassay	PDMS bonded on glass with indium tin oxide patterns	cTnI, cTnT, Myo, BNP	cTnI (0.008 ng/mL), 0.4 ng/mL for cTnT, Myo, BNP	100 s	15 μL^34^
Electrochemical immunoassay	Graphite monolayer on PDMS module	cTnI	1 pg/mL	10 min	15 μL^35^
Centrifugal disks & electrochemical immunoassay	PDMS with TiO_2_ nanofibrous	CRP, cTnI	CRP (286 pg/mL), cTnI (824 pg/mL)	30 min	10 μL^36^
Centrifugal disks & electrochemical immunoassay	Polycarbonate	CRP	4.9 pg/mL	20 min	75 μL^37^
Surface Acoustic Wave	PDMS	CK-MB, CRP, D-dimer, PAPP-A	Less than 1 nM	50 min	not available^38^
Surface plasmon resonance	PDMS bonded on glass	BNP	5 pg/mL	30 min	not available^39^

## Microfluidic-based optical detection

**Fig. 4:**
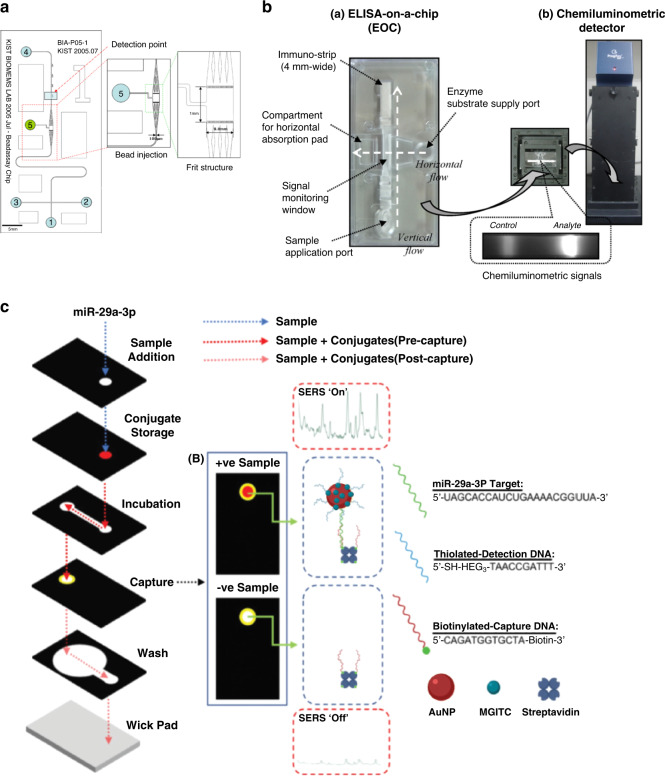
Microfluidic-based optical methods for detecting biomarkers of CVDs. **a** Microfluidic chip for immunofluorescence. **b** Microfluidic system for chemiluminescence immunoassay. **c** Illustration of SERS analysis for miR-29a in the paper-based microfluidic chip. **a** Reproduced with permission^29^. Copyright 2017, Elsevier. **b** Reproduced with permission^31^. Copyright 2017, Elsevier. **c** Reproduced with permission^33^. Copyright 2019, The Royal Society of Chemistry.

Optical detection methods, such as immunofluorescence, chemiluminescence, fluorescence resonance energy transfer (FRET), and surface-enhanced Raman scattering (SERS), are currently the major trend in the detection of CVD biomarkers^80^. Microfluidics has been integrated with these optical detection methods to achieve better diagnosis of biomarkers for CVDs. For instance, a PDMS microfluidic chip embedded with photodiodes and CRP antigen-coated polystyrene beads has been developed to enhance the sensitivity of the miniaturized fluorescence detection of CRP, as illustrated in Fig. 4a. The limit of detection of CRP can be optimized to 1.4 nM, which is 20-fold lower than that of current detection methods^29^. In addition, microfluidic-mediated immunofluorescence has also been applied together with cleavable tag immunoassay (CTI) and micellar electrokinetic chromatography (MEKC) to simultaneously detect and analyze multiple cardiac biomarkers (such as creatine kinase-cardiac muscle isoform (CK-MB), cardiac troponin T (cTnT), cardiac troponin I (cTnI) and myoglobin (Myo)) to diagnose acute myocardial infarction^30^. By labeling the four cardiac biomarker antibodies with different fluorescent substances, the resulting cleaved fluorescent labels can be chromatographically separated and quantitatively measured. The results show that the limits of detection of CK-MB, cTnT, cTnI and Myo can be improved to 3 ng/mL, 25 pg/mL, 2 ng/mL and 5 ng/mL, respectively. The limitation of this system is that CTI and MEKC are carried out separately and cannot be integrated on a single chip. Development of integrated chips that can offer simultaneous on-chip detection of CTI and MEKC would promote the development of tools for the real-time diagnosis of CVDs with great potential for industry transformation.

Chemiluminescence immunoassays are techniques that combine highly sensitive chemiluminescence assays with highly specific immune responses. They can detect more trace amounts of cTnI in blood than conventional immunofluorescence methods. A chemiluminescence diagnostic platform for myocardial infarction was developed by incorporating ELISA into microfluidic chips, as illustrated in Fig. 4b. The proposed chemiluminescence microfluidic chip can save enzymatic reaction time and significantly improve the limit of detection of cTnI to 0.027 ng/mL^31^. However, the disadvantage of this method is that the cost of the detection device for chemiluminescence is high, thus hindering its wide application in clinical trials.

FRET is an energy transfer phenomenon between two fluorescent molecules that are very close and is usually used to detect direct interaction between two protein molecules in the cell. FRET has been combined with a microfluidic chip associated with liquid core waveguide (LCW) technology to achieve high-sensitivity and rapid detection of cTnI. To launch FRET, a donor-labeled Protein A molecule is first bound to a quantum dot and then connected to an acceptor-labeled capture antibody. The results show that the limit of detection of cTnI in phosphate buffer and blood samples can be improved to 32 nM and 50 nM, respectively. The proposed FRET-microfluidic chip shows promise as a diagnostic immunoassay technique with rapid-response capability and high specificity^32^. However, the LCW device used in this method is too complicated to be integrated into products aimed at immediate diagnosis. In addition, surface-enhanced Raman scattering (SERS) has also been integrated with microfluidic chips for more sensitive diagnosis of myocardial infarction. In particular, a paper-based microfluidic chip capable of sensing miR-29a, a microRNA associated with myocardial infarction, using SERS analysis was established, as shown in Fig. 4d^33^. The SERS analysis results show that the limit of detection of miR-29a in the microfluidic channels can be improved to 47 pg/μL with high reproducibility. Therefore, the proposed SERS-integrated microfluidic chips show great potential for future implementation in the diagnosis of myocardial infarction.

Moreover, structural color materials with autonomic regulation capability, such as inverse opal GelMA hydrogel scaffolds, have been developed recently^81^. Cardiomyocytes can be assembled with scaffolds to form engineered cardiomyocyte tissues. When subjected to the autonomic beating process, the cardiomyocytes undergo cell elongation and contraction, while the inverse opal-structured hydrogel substrate exhibits synchronous changes in its structural color. In addition, by integrating structural color materials with microfluidics, the formation of a “self-report heart-on-a-chip” can be achieved, which can offer microphysiological visuality for biological research and drug screening in a simpler and more convenient manner and has great potential in testing cardiomyocyte drugs, studying the growth and differentiation of cells and revealing their core biological principles.

## Microfluidic-based electrochemical detection

**Fig. 5:**
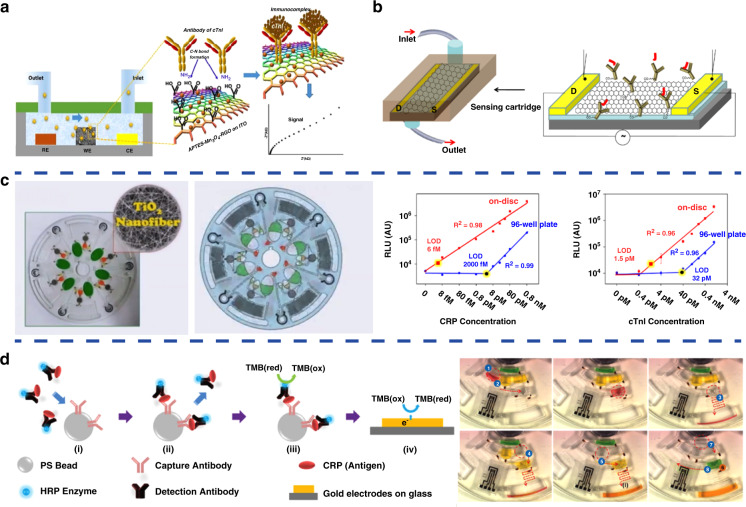
Microfluidic-based electrochemical methods for detecting biomarkers of CVDs. **a** Microfluidic chip nanoengineered with the Mn_3_O_4_-RGO nanocomposite for the detection of cTnI. **b** Single-layer graphite microfluidic chip developed by lithium ion-mediated exfoliation for the detection of cTnI. **c** Centrifugal microfluidic lab-on-a-disc composed of TiO_2_ nanofibers (left) for the detection of CRP and cTnI (right). **d** The fully automated polycarbonate centrifugal microfluidic disc for the detection of CRP. **a** Reproduced with permission^34^. Copyright 2017, American Chemical Society. **b** Reproduced with permission^35^. Copyright 2018, Elsevier. **c** Reproduced with permission^36^. Copyright 2016, MyJoVE Corporation. **d** Reproduced with permission^37^. Copyright 2013, The Royal Society of Chemistry

Compared with optical techniques, microfluidic-based electrochemical detection methods have unique characteristics, such as quick response, high sensitivity, miniaturization of electrodes, and no special requirements for chip materials, with significant progress achieved. For example, an ultrasensitive PDMS microfluidic chip nanoengineered with microporous manganese-reduced graphene oxide (Mn_3_O_4_-RGO) nanocomposites for the detection of cTnI has been developed, as illustrated in Fig. 5a^34^. Due to the large surface area for enhanced loading of antibody molecules and improved electrochemical reaction at the sensor surface, this microfluidic detection chip shows an excellent sensitivity of log [87.58] kΩ/(ng mL^−1^)/cm^2^ for quantification of cTnI over a wide detection range of 0.008–20 ng/mL. Moreover, the chip can also offer highly stable, highly reproducible, and minimal interference detection of other biomarkers, including cTnT, Myo and BNP. In addition, a single-layer graphite microfluidic chip developed by lithium ion-mediated exfoliation has been established and used as a highly sensitive label-free electrochemical detection platform for cTnI, as illustrated in Fig. 5b^35^. The graphite microfluidic chips are functionalized with anti-cTnI antibodies and exhibit excellent sensitivity in the picogram range (~1 pg/mL) for cTnI detection. Compared with optical methods, the detection limit of electrochemical methods is much lower, making them more favorable for detecting rare and precious samples.

In addition, centrifugal microfluidic detection discs have attracted great interest due to advantages such as automation, one-time use, no need for syringe pumps, and the use of only centrifugal force to drive liquid samples with no complicated fluid interactions. For instance, a centrifugal microfluidic lab-on-a-disc composed of TiO_2_ nanofibers has been designed, as shown in Fig. 5c and d^36^. This disc can perform ultrasensitive detection of serum proteins, CRP and cTnI with femtomolar (fM) detection sensitivity from a very small volume of whole blood (10 μL) in 30 min, as demonstrated by the plots in Fig. 5c. Moreover, a fully integrated centrifugal microfluidic disc has been developed with features for target antigen capture from biological samples, as illustrated in Fig. 5d^37^. The antigen in biological samples can be captured through centrifugation and then the fully automated detection process can be completed in less than 20 min. The results show that the limit of detection for CRP can be improved to 4.9 pg/mL, a 17-fold improvement over quantification by optical density. Although centrifugal microfluidic detection discs offer fast and efficient detection, the design and fabrication of the discs are tedious and complicated, which increases the cost of the discs and drastically limits their practical applications.

## Microfluidic-based acoustic detection

Surface acoustic wave (SAW) biosensors have been integrated with microfluidic chips for rapid and efficient analysis of cardiac biomarkers^38^. Multiple cardiac biomarkers, including CK-MB, CRP, D-dimer and pregnancy-associated plasma protein A (PAPP-A), can be efficiently diagnosed with detection limits of less than 1 nM. Moreover, the proposed microfluidic-based SAW chip can also distinguish CRP and PAPP-A from mixtures of the four biomarkers.

## Microfluidic-based NMR and SPR detection

Miniaturized microfluidic chips integrated with nuclear magnetic resonance (NMR) sensors have been developed for multiplexed, quantitative and rapid diagnosis and analysis of cardiac biomarkers^82^. By loading magnetic particles modified with specific antigens as a proximity sensor to amplify molecular interactions, the microfluidic-based NMR platform can generate maximal NMR signals from small volumes (5–10 μL) of biological samples in a short amount of time. Moreover, surface plasmon resonance (SPR) sensors have also been integrated with microfluidic chips to develop portable devices for the efficient detection of BNP^39^. The developed microfluidic-based SPR chips can detect BNP at concentrations as low as 5 pg/mL in 30 min, which drastically improves the limitation of detection and reduces the detection time.

In conclusion, as an emerging technology, microfluidic-mediated detection has the characteristics of low sample demand, portability and fast analysis, making it an ideal detection platform for CVD biomarkers. The application of microfluidics in the diagnosis of CVDs is in line with the development trend of automation and simplification of laboratory medicine to meet the requirements of rapid detection in time for patients with cardiovascular diseases, therefore facilitating more efficient treatment of CVDs.

## Microfluidics for developing efficient therapeutic treatments for CVDs

Due to its ability to finely regulate fluid flow conditions while providing a means for modifying the geometry and surface of microchannels as well as patterning cells in artificial 3D cardiovascular systems, microfluidics shows great advantages for developing CVD treatments, especially for high-throughput drug discovery and developing delivery vehicles.^78,83–85^

## Microfluidic-based cardiovascular drug discovery

Drug therapy is the primary treatment method for CVDs; therefore, accurately evaluating drug efficacy and screening new drugs are of great significance for achieving more efficient clinical treatment.^11,86–88^ By using microfluidic-based cardiovascular-mimetic organs-on-a-chip, including vascular models and heart models, the physiological and pathogenic environment of cardiovascular tissues and organs can be well simulated in vitro, offering great potential for investigating the therapeutic mechanisms of drugs and performing high-throughput compound screening with better efficacy for new drug discovery^22,89^.

**Fig. 6:**
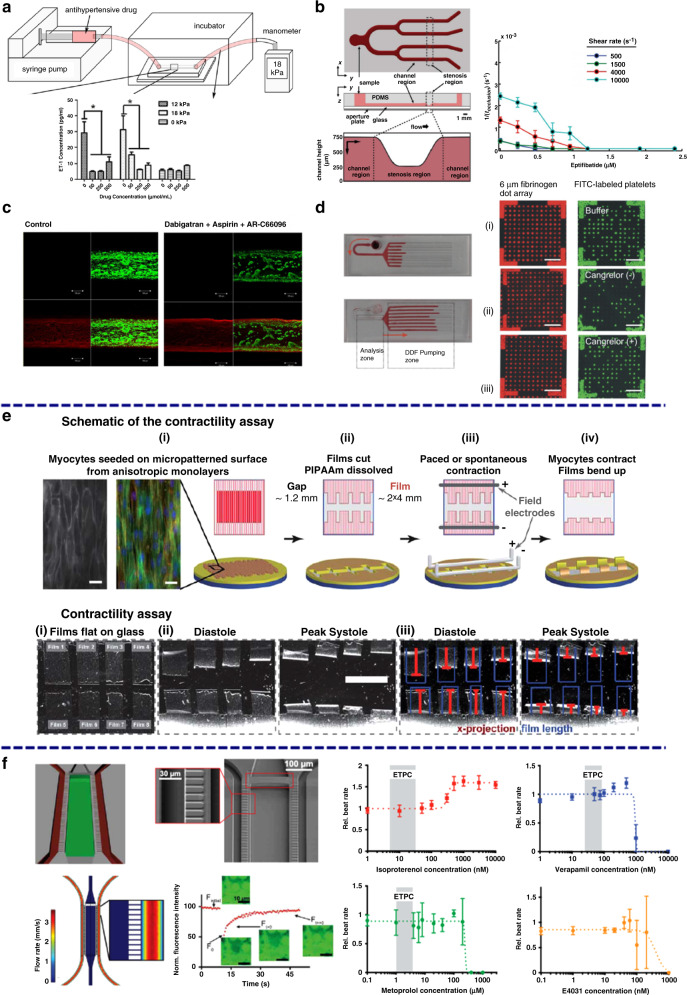
Representative microfluidic-based cardiovascular models for drug discovery. **a** Evaluation of the smoothing effect of hydralazine hydrochloride in hypertension. **b** Investigation of the antiplatelet capacities of aspirin and eptifibatide. **c** Evaluation of the antiplatelet effects of abciximab and cangrelor. **d** Study of the anticoagulant effects of dabigatran and rivaroxaban with aspirin and AR-C66096 as regulatory factors. Microfluidic-based heart models for drug discovery (**e**, **f**). **e** High-throughput evaluation of the inotropic effects of isoproterenol and digoxin. **f** Investigation of the pharmacological properties of verapamil and metoprolol. **a** Reproduced with permission^43^. Copyright 2014, American Chemical Society. **b** Reproduced with permission^90^. Copyright 2014, PLOS. **c** Reproduced with permission^91^. Copyright 2014, PLOS. **d** Reproduced with permission^92^. Copyright 2016, American Chemical Society. **e** Reproduced with permission^95^. Copyright 2011, The Royal Society of Chemistry. **f** Reproduced with permission^47^. Copyright 2015, Springer Nature.

The greatest benefit of using microfluidic-based vascular models in cardiovascular drug discovery is the capability to precisely regulate the fluid flow conditions in microchannels, such as flow rate, shear stress and pulsatile flow, and therefore create pathological and hemodynamic models of CVDs in vitro^63^. For instance, an easily fabricated and assembled microfluidic-based vascular model has been designed to establish an in vitro model of hypertension^43^. As illustrated in Fig. 6a, HUVECs are first cultured in microfluidic channels, and then the antihypertensive drug hydralazine hydrochloride is infused into the channel at different concentrations. By changing the injection speed of fluids in the syringe pump, various atmospheric pressures can be obtained and used to mimic the different hypertension states in the vasculature. The results show that hydralazine hydrochloride can significantly reduce the endothelin-1 level in HUVECs under constant pressure, indicating the concentration-related smoothing effect of hydralazine hydrochloride on hypertension. This platform can be extended to screen drugs with pharmacological effects on hypertension other than hydralazine hydrochloride and may also be used to investigate the underlying action mechanisms of hypotensive drugs at the subcellular level.

Microfluidic-based thrombus models are also used to investigate the antiplatelet effect of antithrombotic drugs. For instance, a microfluidic device with multiple stenotic regions and tunable fluid shear rates was constructed, as shown in Fig. 6b^90^. The antiplatelet capacities of aspirin and eptifibatide have been evaluated by this model. The results show that the two drugs have significant antiplatelet efficacy at high pathological shear rates compared to normal physiological shear rates. Moreover, the effectiveness of aspirin against high shear thrombosis is several orders of magnitude lower than that of eptifibatide. In addition, high shear thrombi formed with aspirin are more likely to detach than those formed without aspirin or with eptifibatide. Eptifibatide reduces the occurrence of occlusion when controlling for shear rate, and its efficacy increases at all shear rates when increasing the concentration. Additionally, a microfluidic chip was designed to simulate the arteriovenous structure to study the anticoagulant effects of dabigatran and rivaroxaban with aspirin combined with AR-C66096 used as a regulatory factor to promote drug interactions^91^. As demonstrated in the CLSM images in Fig. 6c, both dabigatran and rivaroxaban can significantly inhibit thrombosis when their concentrations are increased. The inhibitory effect of the suppository is weakened, and dual application of the antiplatelet drugs can further increase the anticoagulant effect of dabigatran and rivaroxaban. Apart from inducing thrombi by changing the design of the microfluidic channels, surface modifications have also been applied. For instance, fibrinogen is modified on the surface of microchannels and triggers the sedimentation of platelets, as illustrated in Fig. 6d^92^. The binding rate between fibrinogen and platelets was monitored as a signal to evaluate thrombosis in the vasculature. The antiplatelet effects of two representative drugs, abciximab and cangrelor, were systematically investigated. The results show that the two tested drugs indeed show significant antithrombotic efficacy that can be optimized by tuning the practical concentrations. Microfluidic-based thrombus models for antithrombotic drug discovery and evaluation have been well developed, mainly due to the close imitation of cardiovascular hemodynamics by microfluidics. However, most of the presented studies are from the physical perspective, while the treatment of thrombi in vivo should also have a close relationship with vascular endothelial cells. Therefore, the influence of the vascular endothelium should also be taken into account when designing novel models to investigate the antiplatelet effect of antithrombotic drugs.

Recent advances in the field of microfluidics have allowed researchers to construct in vitro heart models for disease modeling and drug testing^93,94^. For example, a microfluidic model in which cardiac myocytes can be seeded was designed and fabricated, as illustrated in Fig. 6e^95^. By adding collagen and fibronectin to the microchannels, cardiac myocytes can form 3D muscular thin films in the device. The device can be further integrated with electrodes and used as a suitable platform for investigating cardiac contractility in vitro. In particular, the positive inotropic effects of isoproterenol and digoxin on cardiac contractility were tested by using the developed model. Moreover, the model can perform high-throughput screening of the inotropic efficacies of drugs with concentrations ranging from 1 nM to 100 mM. In addition, by culturing human induced pluripotent stem cells in microfluidic devices for weeks, they can be induced to form viable and functional cardiac tissues, as illustrated in Fig. 6f^47^. The developed microfluidic heart model can be used as a platform for investigating the pharmacological properties of drugs, such as cardiotoxicity. Specifically, the half maximal inhibitory/effective concentration values (IC_50_/EC_50_) of verapamil and metoprolol determined by the formed MPS are 950 nM and 244 μM, respectively, which are more consistent with the data on tissue scale references compared to cellular scale studies, as demonstrated by the plots in Fig. 6f. This study inspires new perspectives for investigating the pharmacological effects of cardiac drugs at the tissue level or even the organ level. Information obtained at the cellular level is simple and independent, while at the tissue and organ levels, the pharmacological effects of drugs are affected by a number of factors; therefore, the information obtained is more representative and of greater practical significance.

## Microfluidic-based cardiovascular drug delivery vehicles

**Fig. 7:**
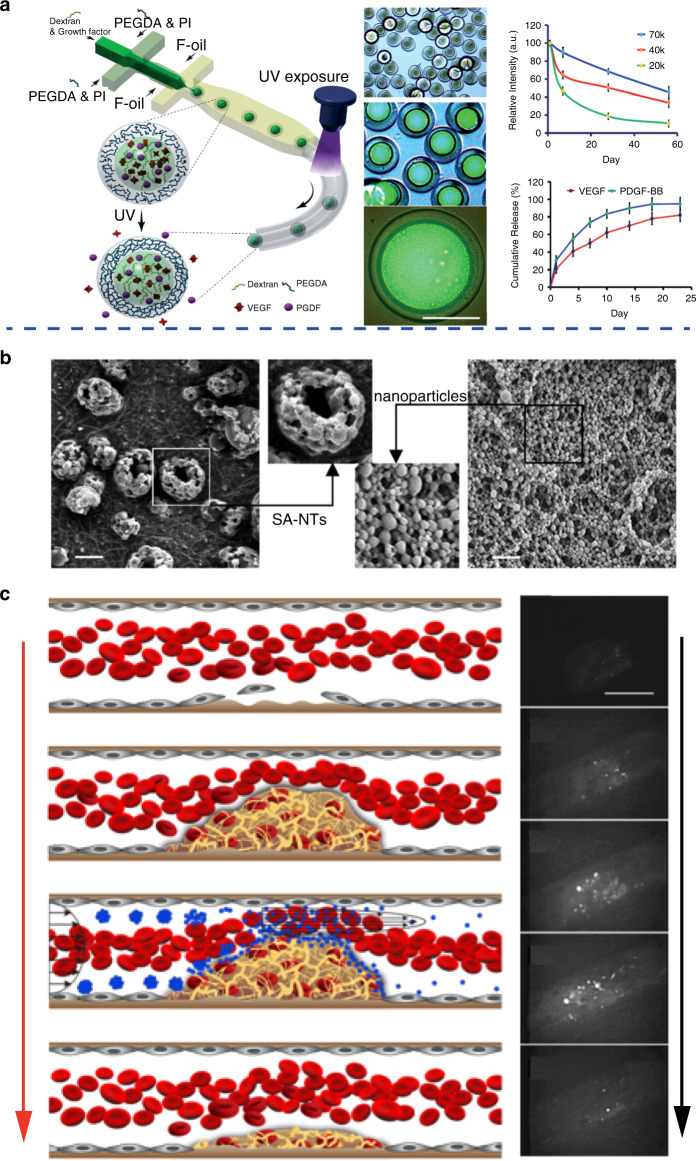
Representative microfluidic-based cardiovascular drug delivery vehicles. **a** Microfluidic fabrication of microcapsules for dual delivery of VEGF and PDGF (left); fabricated microcapsules (middle, scale bar: 50 μm) that can sustainedly release VEGF and PDGF (right). **b** SEM images of the fabricated microaggregates of PLGA nanoparticles, scale bars: 2 μm. **c** Formation of a thrombus with obstructed blood flow and thrombolysis after injection of fabricated microaggregates of PLGA nanoparticles; scale bar: 100 μm. **a** Reproduced with permission^96^. Copyright 2020, The Royal Society of Chemistry. **b**, **c** Reproduced with permission^102^. Copyright 2012, American Association for the Advancement of Science.

Apart from being used for investigating therapeutic mechanisms and high-throughput screening of cardiovascular drugs, microfluidics has also been applied as a novel tool for fabricating advanced vehicles for delivering cardiovascular drugs with superior performance. Various types of vehicles, including nanoparticles, nanocompositions, microneedles, microcapsules and microparticles, have been fabricated, and their capability of delivering cardiovascular drugs has been demonstrated^96–101^. For instance, biocompatible reservoir microcapsules (diameter ∼100 μm) with a large liquid core and polymeric shell have been successfully fabricated through microfluidics, as shown in Fig. 7a^96^. By tuning the phase separation process of poly(ethylene glycol) diacrylate (PEGDA) and dextran, the loading efficiency of vascular endothelial growth factor (VEGF) and platelet-derived growth factor (PDGF) in the microcapsules can be optimized to above 80%. The fabricated microcapsules offered sustained release of VEGF and PDGF for 30 days. Drug-loaded microcapsules can enhance the proliferation of HUVECs and umbilical artery smooth muscle cells (SMCs) as well as subsequent tube formation in vitro. In addition, the ratio of heart tissue scarring was reduced to 11.2% after microcapsule treatment compared with 21.4% after saline treatment in a rat model.

The pathological environment of CVDs can offer inspiration for developing new drug delivery vehicles. For example, taking advantage of the high shear stress caused by vascular narrowing, novel vehicles with targeted drug release capacity to treat thrombosis can be developed. This unique phenomenon can be used as a guideline for developing microaggregates of PLGA nanoparticles that are responsive to shear stress, as demonstrated in Fig. 7b^102^. The fabricated microaggregates can break up into smaller PLGA nanoparticles when subjected to high shear stress and thus release the encapsulated tissue plasminogen activator (tPA). Moreover, an in vitro microfluidic-based thrombosis model was developed and used to investigate the shear stress-responsive breakup of microaggregates. As demonstrated in Fig. 7c, the microaggregates could break up into PLGA nanoparticles and release tPA at thrombotic sites, and significant thrombolysis could be achieved within 5 min after the injection of the tPA-loaded microaggregates. This innovative study opens up new possibilities for taking advantage of the unique pathological conditions of CVDs to design and fabricate novel cardiovascular drug delivery vehicles. Compared with conventional methods, microfluidic-mediated methods can fabricate high-performance vehicles with uniform morphology, size and size distribution, reduced batch-to-batch variation and controllable drug delivery capacity, which is favorable in treating CVDs in a more efficient manner^21^.

## Conclusions and outlook

In conclusion, we have summarized the state of art of microfluidics applied in comprehensive cardiovascular disease research. Various microfluidic-based artificial cardiovascular-mimetic models that can replicate the structural and functional characteristics of their counterparts in nature have been designed and fabricated that show great promise in conducting in-depth and comprehensive investigations of CVDs, including the interpretation of critical pathogenetic mechanisms, the development of more accurate diagnostic methods and the establishment of more practical therapeutic treatments. In addition, microfluidics can also be used as a versatile strategy for fabricating advanced vehicles for efficiently delivering cardiovascular drugs with superior performance. The concept of utilizing microfluidics in cardiovascular disease research further opens up new perspectives for a broader range of disease treatments that can lead to better understanding in physiological and pathological science and propel new advancements in therapeutic techniques and applications. Many emerging opportunities, in addition to their challenges, are briefly discussed below.

### Developing more sophisticated microfluidic-based models for investigating pathogenesis

Although various types of microfluidic-based cardiovascular models have been developed for investigating the pathogenesis of CVDs, the structural and functional complexity of the current models are far from complete and deserve deeper study. The future development of more powerful microfluidic-based models can be carried out at the macro and micro levels. From a macro point of view, current pathogenetic models typically focus on only one type of CVD, such as arteriovenous thrombosis or myocardial infarction; however, the pathological progression of CVDs is a complicated process and always comprises several complications^103,104^. Therefore, it is worth developing more sophisticated models that can be used as suitable platforms for investigating the pathological process of CVDs in a more comprehensive manner. From a micro point of view, microfluidics can be used to develop models for investigating the pathogenesis of CVDs at the subcellular level. Specifically, the biological intracellular environment is heterogeneous and full of distinct compartments with different compositions and properties that perform many key vital processes, including biomolecular localization, signaling, structuring of cytoplasm and the protection of active components in cells, that rely on these organelles^105,106^. Recently, studies of the disruption of these processes and their relationship with the pathogenesis of disease have attracted great interest^107,108^. Therefore, investigation of the pathogenesis of CVDs at the subcellular level would be one of the major trends in the future. All-aqueous microfluidics, an emerging type of microfluidics with superior biocompatibility and high controllability, has been demonstrated to be capable of forming liquid structures that can mimic intracellular structures^109,110^. Therefore, all-aqueous microfluidics has great potential to be applied as in vitro models to investigate the pathogenesis of CVDs more directly at the subcellular level^111^. This concept is mainly in its infancy, and there is much room for further achievements.

### Developing more accurate and convenient microfluidic-based diagnostic methods

Currently, the POC of CVDs is well recognized in the clinic and has become the major trend in the future development of CVD diagnosis^112^. Therefore, microfluidic-based diagnostic methods should be further optimized to fit the requirements of POC tests^113^. For instance, the limitation of detection for biomarkers of CVDs should be further improved, and the detection time should be reduced to offer a more efficient assay process, which requires the further miniaturization of the geometry of microfluidic channels, even to the nanometer level^114^. Moreover, to achieve one-step detection and analyses, microfluidic chips are typically associated with external instruments, such as optical microscopy and computers, which hampers their convenience when used for POC tests. Therefore, a more integrated and portable microfluidic system for CVD biomarker detection is favored in the future development of novel diagnostic methods^115^.

### Developing more biocompatible microfluidic-based drug delivery vehicles

The wide applicability of microfluidics for fabricating materials with well-tailored structures and functions has been demonstrated, with many studies about using microfluidics to fabricate materials as vehicles for delivering cardiovascular drugs reported. Aiming at treating CVDs in vivo, the biocompatibility of fabricated vehicles is the primary issue that needs consideration^116^. Typical microfluidic-assisted fabrication of vehicles always involves the use of organic solvents as the oil phase, and the resultant water-oil interface may lead to the denaturation of delivered drugs. In addition, the residual organic solvents in the fabricated vehicles would also hamper their applications in vivo^117^. Therefore, developing microfluidic-based cardiovascular drug delivery vehicles in a more biocompatible way is desired and worth further investigation.
